# Country/Region Level Pandemic Severity Moderates the Relationships among Risk Experience, Perceived Life Satisfaction, and Psychological Distress in COVID-19

**DOI:** 10.3390/ijerph192416541

**Published:** 2022-12-09

**Authors:** Yi-Hui Christine Huang, Jie Sun, Ruoheng Liu, Jennifer Lau, Qinxian Cai

**Affiliations:** Department of Media and Communication, City University of Hong Kong, Kowloon Tong, Hong Kong

**Keywords:** risk resilience model, social comparison, perceived life satisfaction, country/region-level severity, COVID-19

## Abstract

Scholars and communications practitioners worldwide have sought novel resilience models amid heightened rates of psychological distress caused by the COVID-19 pandemic. We examined perceived life satisfaction as a determinant of resilience. Additionally, we investigated the assumption that perceived pandemic severity at the country/region level moderates structural relationships within our risk–resilience model. Analyzing more than 34,000 valid samples from 15 countries/regions, we found that (1) perceived life satisfaction alleviated psychological distress across all 15 countries/regions; and (2) country/region-level pandemic severity moderated the relationships among COVID-19 symptom experience, perceived life satisfaction, and psychological distress. The effects of COVID-19 symptom experience and perceived life satisfaction on psychological distress were conditional. We discuss possible mechanisms behind our findings and provide practical implications for mitigating psychological distress during public health crises.

## 1. Introduction

Individuals worldwide have reported heightened levels of psychological distress as a result of the risks posed by the COVID-19 pandemic. Since the first confirmed COVID-19 case in December 2019, more than 609 million infections have been recorded globally [1]. COVID-19 caused serious disruptions to public health and economic activity [2,3], with normal daily routines interrupted for large swathes of the global population [4]. Unsurprisingly, pandemic-related health and socio-economic risks led to frequently reported feelings of stress, anxiety, fear, helplessness, and depression [5,6,7,8].

While risk experience can increase negative emotions, protective assets (i.e., external and/or internal factors that help people achieve stability) can offset psychological distress (risk resilience model, [9,10,11]). Individuals with sufficient protective assets demonstrated optimism and active coping styles when experiencing risks [12]. They were more likely to maintain or improve mental health after short disruptions to normal functioning [13,14,15]. 

Perceived life satisfaction is one protective asset that has been proven to mitigate psychological distress [16,17,18]. Perceived life satisfaction is a subjective judgment about the extent to which an individual’s expectations for their own life course have been met [19]. When quality of life matches an individual’s standards, that individual will feel highly satisfied [20]. Empirical studies have verified that a high level of perceived life satisfaction is correlated with lower anxiety and stress in various regions (e.g., Europe [21,22,23], Latin America [24], and Asia [25,26]). However, life satisfaction is not a stable asset; it can be perturbed by life events [27,28]. Scholars demonstrated that exposure to natural disaster, pandemic, and negative life events reduced perceived life satisfaction [29,30]. How has COVID-19 influenced perceived life satisfaction? Is perceived life satisfaction capable of alleviating psychological distress during the pandemic? These questions merited further exploration and formed the basis of our study.

First we asked: does the effect of risk experience and perceived life satisfaction on psychological distress always work? The answer seems to be no. Levels of psychological distress vary from person to person even under identical conditions [31,32]. Social comparison theory elaborated on this phenomenon by proposing that one’s self-judgment depends not only on one’s self-perception, but also on the way in which one perceives others [33]. People search out information on the abilities, attitudes, or achievements of others, and they compare these with their own self-image to better define themselves [34]. In adverse contexts, such as crises or other dangerous situations, investigators observed that risk perception was shaped by social comparison to a high degree [35,36]. When conducting downward comparisons (using referents perceived to be worse off than oneself), people generally perceived themselves to be better off than others and experienced little to no psychological distress as a result [37,38]. By contrast, when conducting upward comparisons (using referents perceived to be better off than oneself), people generally perceived their situation to be worse than most and suffered from unpleasant emotions as a result [39,40].

People use groups or social aggregates to establish a basis for social comparison [41]. These can be specific groups (e.g., friends, neighbors, or colleagues) or general social aggregates (e.g., groups defined by race, socioeconomic class, or nationality) [33,42]. When faced with risk contexts, people seem to prefer aggregated targets of comparison (i.e., to compare themselves to the average) [35,43,44,45]. One major aggregate baseline of social comparison available to the public during the COVID-19 pandemic was overall pandemic severity at the country or regional level. Disease control agencies worldwide reported the number of infections and deaths in their jurisdictions, providing regular public updates on the average level of pandemic exposure in a given country [46]. The aggregate pandemic situation at the country/region level at any given time was therefore readily accessible via government bulletins and news media reports for many different countries and regions. Therefore, our study further proposed that country/region-level severity serves as a reference baseline for social comparison capable of influencing the effects of risk experience and perceived life satisfaction on psychological distress. Specifically, we predicted that our sample would be more likely to demonstrate downward comparisons when country/region-level pandemic severity in other countries was high. The positive self-perceptions that guide such comparisons better protect people from psychological distress. Conversely, we predicted that people would be more likely to conduct upward comparisons when country/region-level pandemic severity in other countries was low. In such instances, risk experience demonstrated stronger impacts on both psychological distress and perceived life satisfaction.

**Figure 1:**
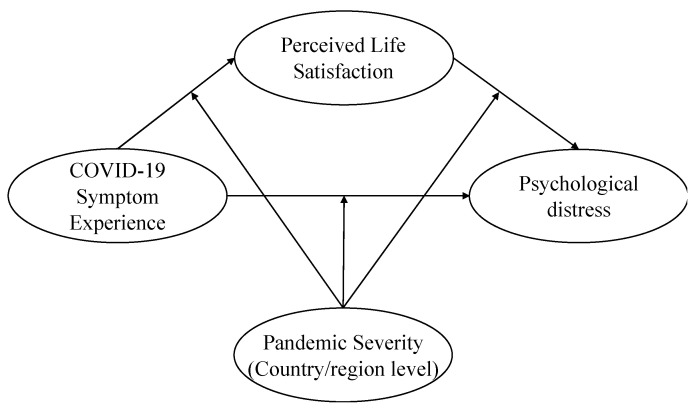
Theoretical framework.

Encountering risk causes psychological distress, but a high level of perceived life satisfaction can offset psychological distress. The effect of risk experience and perceived life satisfaction on psychological distress may be conditional due to social comparison. While some studies have argued that a high level of country/region-level pandemic severity is associated with a high degree of psychological distress [47,48], few studies have focused on the moderating effect of pandemic severity from a social comparison perspective. To investigate the role of social comparison, this study (1) examines the effect of perceived life satisfaction as a protective asset capable of mitigating psychological distress, and (2) explores the moderation effect of country/region-level pandemic severity on the relationships among COVID-19 symptom experience, perceived life satisfaction, and psychological distress (Figure 1 illustrates the theoretical framework).

## 2. Materials and Methods

### 2.1. Data Collection

We used various databases to collect individual-level and country/region-level data in this study. 

Individual-level data came from the Values in Crisis survey (VIC, [49]). Initiated by a group of researchers in Germany, the UK, and Sweden, VIC is a global study project that collected data regarding various value-based and perceptual dimensions during the COVID-19 pandemic. Data were gathered from 17 countries/regions, including eight countries in Europe, two Latin American countries, five Asian countries/regions, and two countries from the Commonwealth of Independent States. Of this larger VIC sample, we used samples from 15 counties/regions, collected primarily during 2020–2021 (the first wave of the VIC survey). We selected these 15 countries/regions for three reasons. First, these countries/regions represent different areas and cultures, enhancing the general applicability of our study. Second, these countries/regions were mostly surveyed in the same year (i.e., 2020), making the data more comparable. Third, these countries/regions provided public COVID-19 pandemic statistics and were, therefore, clearly divisible into groups defined by pandemic severity. See Table A1 for details on the survey period and sample size by country/region. 

We extracted country/region-level data (i.e., pandemic severity by country/region) from the online source “Our World in Data” [1], which publishes statistical data on the COVID-19 pandemic for 207 countries/regions. COVID-19 data published on the site includes the number of confirmed cases, deaths, testing data, vaccination rates, hospitalizations, and more. From this source, we gathered the number of cumulative confirmed cases (per million people) for each country/region.

### 2.2. Measurement

Psychological distress was measured with a version of the Patient Health Questionnaire (PHQ, [50]), modified to include an item about feeling lonely. The VIC survey asked respondents how often (on a scale of 1 to 4, with 4 being very often) they were bothered by the following problems: (1) feeling nervous, anxious, or on edge; (2) not being able to stop or control worrying; (3) feeling down, depressed, or hopeless; (4) having little interest or pleasure in doing things; and (5) feeling lonely. COVID-19 symptom experience was measured by asking participants whether they have or had symptoms of COVID-19. Respondents chose either “yes” or “no”. Borrowing from previous studies of life satisfaction [51,52], the VIC survey measured perceived life satisfaction through four specific dimensions on a scale from 1 to 10: health condition, financial situation, social relations, and work-life balance. An additional question about general life satisfaction was included. 

The number of cumulative confirmed cases is the indicator most frequently used to measure pandemic severity [46,53,54]. We adopted it as the measurement of country/region-level pandemic severity. For each country/region, we recorded cumulative confirmed cases (per million people) on the day the VIC survey started (see Table A2 for detailed dates and numbers). Sample size differences across countries/regions strongly biased results. Consequently, we divided each of the 15 countries/regions into two groups prior to conducting a multigroup analysis. Countries with more cumulative confirmed cases (per million people) than the overall median (1191.49 cases/million people) were placed in a high severity group; those with fewer cumulative confirmed cases were placed in a low severity group. Brazil, assigned the median of 1191.49 cases/million people, was placed in the high severity group. 

**Figure 2:**
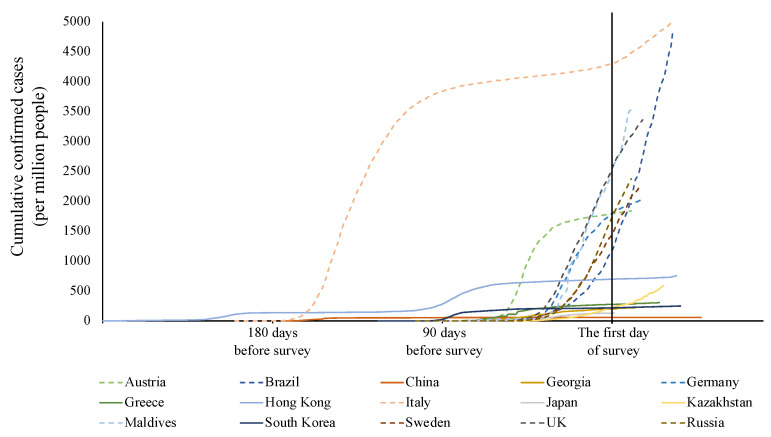
Cumulative confirmed cases (per million people) of studied countries/regions. Figures represent the number of daily cumulative confirmed cases (per million people) from the first reported cases to the final day of the VIC survey in each country/region. The solid lines represent countries/regions in the low severity group. The dotted lines represent countries/regions in the high severity group. The reference line is the first day of the VIC survey in each country/region.

Peak severity occurred at different points in time in different countries/regions. We, therefore, compared daily cumulative confirmed cases (per million people). Extreme changes in severity may have biased assignment to one or the other severity group. As shown in Figure 2, countries/regions in the high severity group showed sharp growth in cumulative confirmed cases before the survey, and confirmed cases continued to increase during the survey period. Counties/regions in the low severity group showed relatively steady growth in cumulative confirmed cases. Overall, our severity group assignments were reasonable.

### 2.3. Statistical Analysis 

We used SmartPLS (v. 3.3.3, SmartPLS GmbH Company, Oststeinbek, Germany) to assess structural relations among variables. We applied multi-group analysis (MGA) to compare the high and low severity groups. MGA is an efficient approach to evaluating moderation across multiple relationships in a research model [55]. Demographic factors including gender, age, education, and income were established as covariates to control for potential extraneous effects. After deleting samples with missing independent and dependent variable values, we used the mean replacement method [56] to fill in missing values for control variables.

## 3. Results

### 3.1. Descriptive Statistics

As shown in Table A3, the dataset consisted of 34,005 valid samples, comprising 16,438 males (48.4%) and 17,550 females (51.6%). Over half the participants (53.3%, *n* = 18,130) were between 20 and 50 years old. Fifty-five percent of respondents (*n* = 18,818) reported high education levels (received at least short-cycle tertiary education). Roughly one-third of the sample reported income at a quarter of the global average (27.9%, *n* = 9117). Worldwide, on average, our dataset did not report high levels of psychological distress (M = 1.75, SD = 0.76). Respondents across the dataset indicated their perceived life satisfaction was not bad (M = 6.28, SD = 1.93). Most participants (90.8%, *n* = 30,877) reported having never experienced COVID-19 symptoms. Few participants (9.2%, *n* = 3128) have or had symptoms. 

The high severity group included 7696 males (48.7%) and 8107 females (51.3%). Almost half (48.5%, *n* = 7676) were between 20 and 50 years old. Thirty-five percent of this group (*n* = 5607) reported high education levels (received at least short-cycle tertiary education). Over one-third of the high severity group reported income at a quarter of the global average (37.1%, *n* = 5785). Participants in this group expressed slightly lower levels of psychological distress (M = 1.73, SD = 0.77) than the overall dataset but also demonstrated a high degree of perceived life satisfaction (M = 6.48, SD = 2.00). A strong majority of high-severity-group participants (85.7%, *n* = 13,557) reported never having experienced symptoms of COVID-19. Few participants in this group (14.3%, *n* = 2261) have or had symptoms of COVID-19. 

The low severity group included 8742 males (48.1%) and 9443 females (51.9%). Over half of the participants in this group (57.5%, *n* = 10,535) were between 20 and 50 years old. Seventy-three percent (*n* = 13,211) reported high education levels (received at least short-cycle tertiary education). Roughly a third of respondents in this group reported high income (32.0%, *n* = 5462). The low severity group expressed slightly higher rates of psychological distress than the dataset as a whole (M = 1.78, SD = 0.76). Further, the low severity group demonstrated a high degree of perceived life satisfaction (M = 6.10, SD = 1.84), with 95.2% participants (*n* = 17,320) reporting never having had COVID-19 symptoms. Very few participants in this group (4.8%, *n* = 867) have or had symptoms of COVID-19. 

### 3.2. Model Quality

We conducted reliability and validity tests to double-check our measurements. Internal reliability was measured using Cronbach’s alpha and composite reliability (CR). All Cronbach’s alpha values were above the 0.7 thresholds [53]. CR values were well above the 0.7 benchmarks [57]. Convergent validity was measured using factor loading and average variance extracted (AVE). Results indicated that all factor loadings in the model were above 0.7, and the AVE values of all study constructs were above 0.5 (See Table 1). Discriminant validity met the Fornell-Larcker criterion [58] (Table 2). The variance inflation factor (VIF) ranged from 1.76 to 2.94 and was well below the threshold of 10 [59,60]. This shows that the model had no collinearity issues. 

The global applicability of the model was evaluated using the normed fit index (NFI) and standardized root-mean-squared residual (SRMR). Relational structures in all samples were supportive of the proposed model (full sample: NFI: 0.92, SRMR: 0.04; high severity group: NFI: 0.90, SRMR: 0.05; low severity group: NFI: 0.94, SRMR: 0.04).

### 3.3. Path Effect

As Table 3 illustrates (full sample), COVID-19 symptom experience has a significant positive relationship to psychological distress (β = 0.06, *p* < 0.001). Moreover, perceived life satisfaction negatively affects psychological distress (β = −0.29, *p* < 0.001). However, COVID-19 symptom experience did not demonstrate a significant effect on perceived life satisfaction (β = 0.01, *p* = 0.139). Whether a person has COVID-19 symptoms does not affect their emotional outcomes. 

### 3.4. Multi-Group Analysis

**Figure 3:**
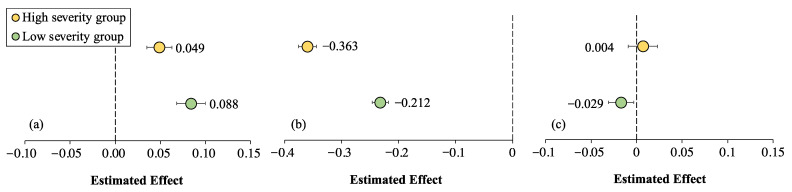
The effect of (**a**) COVID-19 symptom experience and (**b**) perceived life satisfaction on psychological distress and (**c**) the effect of COVID-19 symptom experience on perceived life satisfaction.

Relationships among COVID-19 symptom experience, perceived life satisfaction, and psychological distress were tested across the two severity groups using multi-group analysis. As indicated in Table 3, significant differences emerged between the two groups. The effect of COVID-19 symptom experience on psychological distress in countries/regions with high levels of pandemic severity was stronger than in countries/regions with low severity (*p* < 0.001) (Figure 3a). The effect of perceived life satisfaction on psychological distress in countries/regions with a high level of pandemic severity was weaker than in countries/regions with low severity (*p* < 0.001) (Figure 3b). The effect of COVID-19 symptom experience on perceived life satisfaction was not significant in high pandemic-severity contexts. Nevertheless, symptom experience demonstrated negative effects on perceived life satisfaction in low pandemic-severity contexts (*p* = 0.003) (Figure 3c). Country/region-level severity, therefore, played a moderating role in relationships among COVID-19 symptom experience, perceived life satisfaction, and psychological distress.

## 4. Discussion

This study examined the role of COVID-19 symptom experience and perceived life satisfaction on psychological distress in 15 countries/regions during the COVID-19 pandemic. What is more, by proposing that country/region-level pandemic severity can serve as a baseline for social comparison, this study reveals the conditional effect of COVID-19 symptom experience and perceived life satisfaction on psychological distress. We found that perceived life satisfaction served as a protective factor capable of alleviating pandemic-related psychological distress in all 15 countries/regions. Further, country/region-level pandemic severity moderated relationships among COVID-19 symptom experience, perceived life satisfaction, and psychological distress. Specifically, (1) when pandemic severity was high in a country/region, COVID-19 symptom experience had a relatively minor impact on psychological distress. On the contrary, when a country/region’s pandemic severity was low, individuals reporting symptom experience were more likely to suffer from psychological distress; (2) when infection and mortality rates were high, perceived life satisfaction effectively mitigated some of the pandemic’s negative mental health outcomes. By contrast, when infection and mortality rates were low, perceived life satisfaction had a relatively weak mitigating effect on pandemic-related psychological distress; and (3) when pandemic severity was high, COVID-19 symptom experience did not affect perceived life satisfaction. When mortality and infection rates were low, however, COVID-19 symptom experience significantly reduced perceived life satisfaction.

### 4.1. Perceived Life Satisfaction as a Protective Factor

COVID-19 symptom experience was widely reported to induce psychological distress in the form of anxiety, fear, and loneliness. Simultaneously, perceived life satisfaction consistently proved capable of alleviating emotional suffering. This finding is consistent with previous studies that demonstrated the role of perceived life satisfaction as a protective factor [21,22,23,24,25,26]. In addition, our study provides evidence of different pandemic severity conditions. Although the effect is different depending on the level of pandemic severity, perceived life satisfaction consistently offsets negative emotions related to COVID-19.

Contrary to previous studies, we found that COVID-19 symptom experience did not significantly influence perceived life satisfaction across all groups. The negative correlation between symptom experience and perceived life satisfaction was significant when pandemic severity was low, but non-existent when pandemic severity was high. This is likely due to the tendency of people to evaluate their status using not only their self-perception, but also the way in which they perceive others (i.e., the social comparison process, [34]). The following sections elaborate how country/region-level pandemic severity serves as a moderator via social comparison.

### 4.2. Country/Region-Level Pandemic Severity as a Moderator via Social Comparison

Consistent with our hypothesis, we found that relationships among symptom experience, perceived life satisfaction, and psychological distress were moderated by country/region-level pandemic severity. If an entire country/region was in the grip of a severe pandemic, people perceived all of their fellow citizens to be exposed to great health risks. Relative to this general perception, they perceived their own situation to be relatively acceptable. Whether an individual reported COVID-19 symptoms had no influence on perceived life satisfaction in such cases. At the same time, personal symptom experience no longer led to strong negative emotions. Due to the limited effects of specific experiences on self-perception, perceived life satisfaction, partly determined by personal traits and attitudinal factors [61,62], had a more substantial impact on emotional outcomes in such contexts. If a country or region’s pandemic severity was relatively mild, people tended to view their fellow citizens as being more or less free from risk. In these cases, symptom experience caused high levels of distress and reduced the evaluation of their general life satisfaction. At the same time, mild pandemic conditions were associated with weaker effects of perceived life satisfaction on emotional outcomes.

These findings align with social comparison theory, which holds that people make judgments about their own well-being based on observations of other people [58]. Downward comparison leads people to interpret their situations in a more positive way [37,38]. By contrast, when conducting upward comparisons, people regard their own situation as inferior [40], ultimately experiencing more negative mental health outcomes. Previous studies found that social comparison can moderate the relationship between social activity and negative emotions [63,64]. Our study proved that, in risk contexts, social comparison is also an important psychological process. In general, the condition of the reference group influences one’s self-perception and psychological patterns.

### 4.3. Theoretical and Practical Implications

This study contributes to current research on the risk resilience model. By examining the effect of perceived life satisfaction and protective assets on psychological distress during the COVID-19 pandemic across 15 countries/regions, we extended existing findings. Moreover, by revealing the moderating effects of country/region level pandemic severity via social comparison, this study demonstrated the conditional effect of personal risk experience and protective assets on emotional outcomes. It provides a departure point for us to understand people’s psychological mechanisms in the risk context from a social comparison perspective.

Practically, our study offers insights drawn from the COVID-19 pandemic for psychological mitigation of negative risk perception. Perceived life satisfaction is an effective protective measure across different countries/regions. People with low levels of perceived life satisfaction are more in need of psychological aids during risk scenarios. Risk contexts require interventions aimed at bolstering and maintaining perceived life satisfaction among targeted groups of constituents with reported levels of low life satisfaction [47]. 

Additionally, pandemic severity is an important factor to account for when understanding the public’s psychological status. When faced with high country/region-level pandemic severity, improving the public’s self-perception is an effective response strategy. Utilizing and maximizing the role of protective assets can help people recover from negative psychological outcomes [65]. When pandemic conditions are mild, people need specific guidelines about how to respond to potential risks [66]. By making risk more concrete and familiar, risk managers can reduce the influence of risk exposure [67]. Vaccine hesitancy is another impediment to the fight against COVID-19 that varies across countries or regions [68]. Varying vaccine hesitancy rates across countries/regions may be attributed partly to differences in demographics or context-specific factors [69]. Studies indicated that policymakers at the national level should dive into the local context to overcome vaccine hesitancy [68,69]. Our study found that when country/region level pandemic severity is low, personal symptom experience has a stronger influence on the public’s risk perception. This may explain differences in vaccine hesitancy levels across countries/regions. The moderating effect of country/region level pandemic severity should be considered in future studies that seek to improve vaccination efforts.

## 5. Conclusions

This study revealed the effects of symptom experience and perceived life satisfaction on psychological distress during the COVID-19 pandemic. Country/region-level severity served as a comparative baseline moderating relationships among symptom experience, perceived life satisfaction, and psychological distress. In addition to contributing to the growing body of the literature highlighting the role of protective factors in risk scenarios, these findings also indicated the influence of social comparison on emotional outcomes.

It should be noted that this study has some limitations. First, we only used personal symptom experience as a risk factor. Future studies should take into account more variables capable of heightening risk perception. Second, we divided the 15 countries/regions into two groups based on the median pandemic severity score for all countries/regions. The final grouping may be biased by our sample selection. Future studies should use other research designs (e.g., experiments) to further examine these findings. Third, although public statistics provide global comparative data, proof of their reliability requires further investigation. 

Despite these limitations, our study provides an adequate departure point for future studies to focus on the conditional effects of other risk scenarios and protective factors (e.g., self-control) from a social comparison perspective.

## Figures and Tables

**Table 1:** Measurement reliability result.

	Cronbach’s Alpha	Composite Reliability (CR)	Average Variance Extracted (AVE)
**Full sample**			
Perceived life satisfaction (PLS)	0.87	0.20	0.66
Psychological distress (PD)	0.89	0.92	0.70
**High severity group**			
Perceived life satisfaction (PLS)	0.84	0.89	0.62
Psychological distress (PD)	0.88	0.91	0.68
**Low severity group**			
Perceived life satisfaction (PLS)	0.89	0.92	0.70
Psychological distress (PD)	0.90	0.93	0.71

**Table 2:** Correlations and square roots of AVE values.

	Full Sample *n* = 34,005			High Severity Group *n* = 15,818			Low Severity Group *n* = 18,187		
	PD	SE	PLS	PD	SE	PLS	PD	SE	PLS
Psychological distress (PD)	**0.83**			**0.82**			**0.84**		
COVID-19 symptom experience (SE)	0.07	**1.00**		0.05	**1.00**		0.10	**1.00**	
Perceived life satisfaction (PLS)	−0.30	0.01	**0.81**	−0.40	0.01	0.79	−0.20	−0.01	**0.84**

Note: The bold numbers are square roots of AVE values.

**Table 3:** Multi-group analysis results.

Path	Full Sample *n* = 34,005		High Severity Group *n* = 15,818		Low Severity Group *n* = 18,187		High Severity Group vs. Low Severity Group
	β	CI	β	CI	β	CI	*p* value
COVID-19 symptom experience → Psychological distress	0.057	(0.047, 0.067)	0.049	(0.035, 0.064)	0.088	(0.073, 0.103)	0.000
Perceived life satisfaction → Psychological distress	−0.293	(−0.304, −0.282)	−0.363	(−0.379, −0.348)	−0.212	(−0.228, −0.197)	0.000
COVID-19 symptom experience → Perceived life satisfaction	0.008	(−0.002, 0.019)	0.004	(−0.011, 0.018)	−0.029	(−0.044, −0.013)	0.003

Note: Age, gender, education, and monthly income were controlled.

## Data Availability

Values in Crisis Survey data (SUF edition). Available online: https://data.aussda.at/dataset.xhtml?persistentId=doi:10.11587/LIHK1L (accessed on 9 November 2022). Coronavirus Pandemic (COVID-19) data. Available online: https://ourworldindata.org/covid (accessed on 9 November 2022).

## Appendix A

**Table A1:** Survey period and sample size by country/region.

Country	Fieldwork Period	Sample Size
Austria	14 May 2020–14 May 2020	*n* = 2018
Brazil	18 May 2020–19 June 2020	*n* = 3534
China	15 July 2020–31 August 2020	*n* = 3200
Georgia	28 May 2020–9 June 2020	*n* = 882
Germany	24 April 2020–10 May 2020	*n* = 2009
Greece	23 May 2020–17 June 2020	*n* = 456
Hong Kong	20 October 2020–23 November 2020	*n* = 3061
Italy	Phase 1: 18 August 2020–2 November 2020 Phase 2: 7 January 2021–26 January 2021	*n* = 1378
Japan	15 May 2020–16 May 2020	*n* = 3000
Kazakhstan	5 May 2020–1 June 2020	*n* = 1035
Maldives	23 May 2020–2 June 2020	*n* = 977
Russia	14 May 2020–24 May 2020	*n* = 1306
South Korea	Phase 1: 24 June 2020–1 July 2020 Phase 2: 26 May 2020–2 June 2020	*n* = 6544
Sweden	20 April 2020–5 May 2020	*n* = 2554
United Kingdom	29 April 2020–15 May 2020	*n* = 2033
Total		*n* = 35,428

**Table A2:** Country/region-level severity.

Group	Country/Region	Cumulative Confirmed Cases (per Million People)	Extraction Date
High-severity group	Italy	4298.36	18 August 2020
	United Kingdom	2565.16	29 April 2020
	Maldives	2517.94	23 May 2020
	Germany	1802.97	24 April 2020
	Austria	1788.93	14 May 2020
	Russia	1738.39	14 May 2020
	Sweden	1444.91	20 April 2020
	Brazil	1191.49	18 May 2020
Low-severity group	Hong Kong	701.97	20 October 2020
	Greece	636.82	23 May 2020
	Kazakhstan	289.98	05 May 2020
	Korea	275.34	26 May 2020
	Georgia	219.05	28 May 2020
	Japan	217.34	15 May 2020
	China	196.38	15 July 2020

**Table A3:** Sample distribution.

Category	Full Sample *n* = 34,005		High Severity Group *n* = 15,818		Low Severity Group *n* = 18,187	
	Mean	SD	Mean	SD	Mean	SD
Psychological distress	1.75	0.76	1.73	0.77	1.78	0.76
Perceived life satisfaction	6.28	1.93	6.48	2.00	6.10	1.84
	Frequency	Percentage	Frequency	Percentage	Frequency	Percentage
COVID-19 symptom experience						
no symptom	30,877	90.8	13,557	85.7	17320	95.2
have/had symptom	3128	9.2	2261	14.3	867	4.8
Gender						
Male	16,438	48.4	7696	48.7	8742	48.1
Female	17,500	51.6	8107	51.3	9443	51.9
Age						
<20	681	2.0	501	3.2	180	1.0
20–25	2962	8.7	1595	10.1	1367	7.5
25–30	3815	11.2	1548	9.8	2267	12.5
30–35	3779	11.1	1719	10.9	2060	11.3
35–40	3549	10.4	1440	9.1	2109	11.6
40–45	4025	11.8	1374	8.7	2651	14.6
45–50	3671	10.8	1481	9.4	2190	12.0
50–55	3167	9.3	1265	8.0	1902	10.5
>55	8353	24.6	4895	30.9	3458	19.0
Education						
Low education	5428	16.0	4952	31.3	476	2.6
Medium education	9662	28.5	5242	33.2	4420	24.4
High education	18,818	55.5	5607	35.5	13211	73.0
Income Quartiles						
Income level 1	9117	27.9	5785	37.1	3332	19.5
Income level 2	8484	26.0	4110	26.4	4374	25.6
Income level 3	7404	22.7	3748	22.3	3926	23.0
Income level 4	7675	23.5	2213	14.2	5462	32.0

Note: Low education: lower than secondary education; Medium education: upper secondary education and post-secondary education; High education: higher than short-cycle tertiary education; Income level was divided according to quartiles for global average monthly income (calculated by VIC team).
